# Inconsistency of AI in intracranial aneurysm detection with varying dose and image reconstruction

**DOI:** 10.1038/s41598-025-04830-7

**Published:** 2025-06-06

**Authors:** Leonie Goelz, Angelo Laudani, Ulrich Genske, Michael Scheel, Georg Bohner, Hans-Christian Bauknecht, Sven Mutze, Bernd Hamm, Paul Jahnke

**Affiliations:** 1 Department of Radiology and Neuroradiology, Unfallkrankenhaus Berlin, Warener Str. 7, 12683 Berlin, Germany; 2 Department of Diagnostic Radiology and Neuroradiology, Universitätsmedizin Greifswald, Ferdinand-Sauerbruch-Straße, 17475 Greifswald, Germany; 3 Department of Radiology, Charité – Universitätsmedizin Berlin, Corporate Member of Freie Universität Berlin, Humboldt-Universität zu Berlin, Berlin Institute of Health, Charitéplatz 1, 10117 Berlin, Germany; 4 Department of Neuroradiology, Charité – Universitätsmedizin Berlin, Corporate Member of Freie Universität Berlin, Humboldt-Universität zu Berlin, Berlin Institute of Health, Charitéplatz 1, 10117 Berlin, Germany; 5 Berlin Institute of Health (BIH), Anna-Louisa-Karsch-Str. 2, 10178 Berlin, Germany

**Keywords:** Intracranial aneurysm, Artificial intelligence, Computed tomography angiography, Reproducibility of findings, Imaging phantoms, Medical imaging, Diagnostic markers, Hardware and infrastructure, Image processing, Machine learning, Applied physics, X-rays, Optical sensors, Imaging and sensing

## Abstract

Scanner-related changes in data quality are common in medical imaging, yet monitoring their impact on diagnostic AI performance remains challenging. In this study, we performed standardized consistency testing of an FDA-cleared and CE-marked AI for triage and notification of intracranial aneurysms across changes in image data quality caused by dose and image reconstruction. Our assessment was based on repeated examinations of a head CT phantom designed for AI evaluation, replicating a patient with three intracranial aneurysms in the anterior, middle and posterior circulation. We show that the AI maintains stable performance within the medium dose range but produces inconsistent results at reduced dose and, unexpectedly, at higher dose when filtered back projection is used. Data quality standards required for AI are stricter than those for neuroradiologists, who report higher aneurysm visibility rates and experience performance degradation only at substantially lower doses, with no decline at higher doses.

**Supplementary Information:**

The online version contains supplementary material available at 10.1038/s41598-025-04830-7.

## Introduction

Intracranial aneurysms (IAs) affect approximately 2–3% of the global population^1–3^. While many remain asymptomatic, rupture can lead to devastating consequences such as disablement or death. Computed tomography angiography (CTA) plays a key role in diagnosing IA due to its speed, availability, non-invasiveness, and diagnostic accuracy^4,5^. While traditionally interpreted by radiologists, artificial intelligence (AI)-enabled software tools show promise in enhancing aneurysm detection and management^6,7^.

The U.S. Food and Drug Administration (FDA) has cleared several AI-enabled applications for managing IA based on CTA. However, clearance of these applications does not guarantee reliable model performance in actual clinical use^8^. Variations in CT scanners, protocols, and reconstruction methods in specific clinical practice may differ from those during development, implementation, and validation of AI models, potentially affecting data quality. It remains uncertain whether these applications can reliably perform on such varied data types.

Current methods for independent AI validation involve publicly released datasets as benchmarks to assess algorithm performance^9,10^. These initiatives enable evaluation outside the developmental environment, offering an independent estimate of performance and generalizability^11^. However, this information does not indicate how the algorithm will perform in a specific radiology facility. Furthermore, if imaging systems or protocols are modified, it remains uncertain whether the AI will still perform as expected^12^. Even without apparent modifications, the performance of AI algorithms is prone to decay and benefits from regular monitoring^13^. Addressing these issues requires iterative local validation steps in the specific imaging department^14^. However, each of these iterations requires the collection of patient data, which is time-consuming and impractical for efficiently validating and optimizing scanners, data quality, and AI functionality.

In contrast, phantoms featuring human tissue and pathology patterns enable controlled access to scan data and provide opportunities for standardized consistency testing of AI. In this study, a phantom simulating a patient with three IAs was used to evaluate a diagnostic AI algorithm for intracranial aneurysm detection. We hypothesized that variations in data quality can influence AI output, and that such variations may have a different impact on AI than on image interpretation by neuroradiologists. Therefore, the aim of our study was to assess the consistency of an FDA-cleared and CE-marked AI solution for IA detection across changes in image data quality caused by dose variations and image reconstruction.

## Methods

### Study design

The study was conducted prospectively from April to July 2024 following ethics approval by the ethics committee of the Charité. A CT phantom simulating a patient with three intracranial aneurysms was examined using 21 doses and two reconstruction algorithms. Aneurysm detection was evaluated using a commercial AI solution and by five neuroradiologists.

### Phantom

**Fig. 1:**
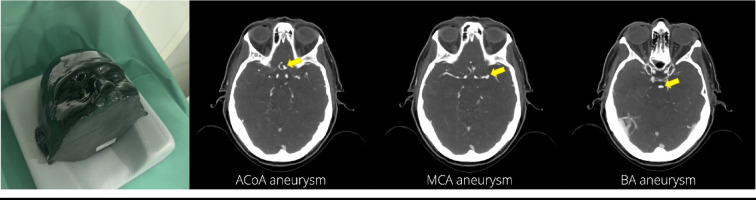
Setup and CT images of the phantom. Photograph shows the phantom positioned in the CT scanner for image acquisition. CT images of the phantom visualize the three aneurysms in the anterior communicating artery (ACoA), middle cerebral artery (MCA), and basilar artery (BA).

A realistic head phantom (50 − 03, PhantomX, Berlin, Germany) specifically designed for AI evaluation was employed in this study. The phantom simulated a patient post contrast medium injection in arterial phase and featured three intracranial aneurysms of the anterior communicating artery (ACoA), middle cerebral artery (MCA), and basilar artery (BA) with maximum diameters of 4 mm, 4 mm, and 2 mm, respectively. It was fabricated using a technology based on previously published methods^15,16^. These methods were specifically developed to replicate human tissues with high fidelity and have previously been shown to accurately reproduce anatomy and pathology^17,18^. The phantom was selected to include saccular aneurysms with varying levels of complexity for evaluating dose and image reconstruction effects on detection: two larger aneurysms, one in the MCA and another in the ACoA near the skull base adjacent to bone, and a smaller, less common BA aneurysm. For image acquisition, the phantom was positioned within a support pillow in the isocenter of the CT scanner gantry reflecting the typical positioning during clinical routine. Figure 1 shows the phantom setup and CT images of the phantom visualizing the three aneurysms.

### Image acquisition

Images were obtained using a 128-row CT scanner (Somatom AS, Siemens Healthineers, Erlangen, Germany). The tube voltage was 120 kVp, the pitch was 0.9, the image matrix was 512 × 512 pixels and the pixel size ranged between 0.39 and 0.42 mm (median: 0.41 mm). Twenty-one computed tomography dose indices (CTDI) ranging from 0.47 to 20.09 mGy were employed. To this end, the tube current and rotation time were varied as detailed in Table 1. Images were reconstructed using the department’s standard iterative reconstruction (IR) algorithm Sinogram Affirmed Iterative Reconstruction (SAFIRE, I26f kernel) and filtered back projection (FBP, B26f kernel). Three acquisitions were performed per dose and image reconstruction, totaling 126 scans. Images were reconstructed in axial orientation with 0.75 mm slice thickness.

**Table 1:** Computed tomography dose indices (CTDIvol), rotation times, and tube current-time products used for image acquisition.

CTDIvol (mGy)	Tube voltage (kVp)	Tube current-time product (mAs)	Rotation time (s)	Slice thickness (mm)
0.47	120	7	0.3	0.75
1.01	120	15	0.5	0.75
2.02	120	30	0.5	0.75
3.00	120	44	0.5	0.75
4.01	120	59	0.5	0.75
5.02	120	74	0.5	0.75
6.03	120	89	0.5	0.75
7.05	120	104	0.5	0.75
8.06	120	119	0.5	0.75
9.07	120	134	0.5	0.75
10.08	120	149	0.5	0.75
11.09	120	164	0.5	0.75
12.11	120	179	0.5	0.75
13.04	120	193	0.5	0.75
14.06	120	208	0.5	0.75
15.07	120	223	0.5	0.75
16.04	120	238	0.5	0.75
17.05	120	253	0.5	0.75
18.07	120	268	0.5	0.75
19.08	120	283	0.5	0.75
20.09	120	298	0.5	0.75

### AI image assessment

The study site had a commercial, cloud-based AI solution (AIDOC, Tel Aviv, Israel) integrated into the clinical workflow, which included algorithms for detection of IA and large vessel occlusion in CTA, and intracranial hemorrhage in non-contrast head CT. The product was proprietary, and access was limited to standard clinical use, with no further insight into its internal mechanisms or operating principles. The IA algorithm was FDA-cleared for flagging and communicating aneurysms above 5 mm size^19^. At the study site, it was used under CE marking and implemented in the year preceding the study to analyze and respond to images without size restrictions. The solution was set up so that CTAs were automatically processed starting with their export to a virtual machine within the department. Here, the software’s orchestrator identified the examined body part and study protocol to ensure processing by the appropriate algorithms. In addition, the orchestrator conducted an initial check for technical issues rendering scans unsuitable for analysis, such as excessive motion artifacts, metal artifacts, inadequate field of view, or insufficient arterial contrast. When the initial check was passed, CTAs with positive IA findings were highlighted in the cloud for prioritization, and all results (positive or negative) were fed back into the picture archiving and communication system as color-coded heatmaps or negative summary reports. The heatmaps and summary reports resulting from the phantom examinations were independently reviewed by two board-certified radiologists with 10 years of experience to determine positive and negative aneurysm labels, followed by a consensus reading.

### Reader image assessment

Five board-certified neuroradiologists participated in a reading experiment to evaluate aneurysm visibility and image quality. Their experience ranged from 9 to 24 years (median: 17 years). Each reader was presented with all 126 datasets in random order and asked to indicate the visibility of each of the three aneurysms (1 = yes, 2 = no). In addition, readers were asked to evaluate image quality for the assessment of each aneurysm (3 = good, 2 = intermediate, 1 = poor). No consensus reading was done. Readers were familiarized with the experimental setup in a training session preceding the reading experiment. All readings were performed using the open-source platform *Human Observer Net*^20^.

### Data analysis

True positive aneurysm labels are presented in absolute numbers. For the analysis of label sizes and intensities, aneurysm labels had to be segmented. To this end, a pixel value of 100 in the blue channel of the 8-bit RGB (red, green, blue) color-coded heatmaps was defined as the maximum threshold for positive aneurysm labels. Label sizes were calculated by multiplying the number of positive pixels by the pixel size. For the analysis of pixel intensities, RGB values of labels were converted into grayscale values, inversed, normalized, and then averaged. Average label sizes and intensities across three repeated acquisitions were compared between aneurysms using the Mann-Whitney U-test and Bonferroni correction to adjust p-values for multiple comparisons. Reproducibility was analyzed using the coefficient of variation (CV), employing only results from those doses and reconstructions with three positive labels. Aneurysms rated as visible by readers are presented in absolute numbers. Image quality ratings of 1 (poor) were assigned the value 0 if readers also rated the aneurysm as not visible. Image quality ratings were averaged per aneurysm and reader across three repeated CT examinations per dose and image reconstruction method. Mean values of all five readers were used to compare image quality ratings between aneurysms and reconstruction methods using the Mann-Whitney U-test with Bonferroni correction of p-values. Differences were interpreted as significant for *p* < 0.05.

## Results

### Imaging and AI output

**Fig. 2:**
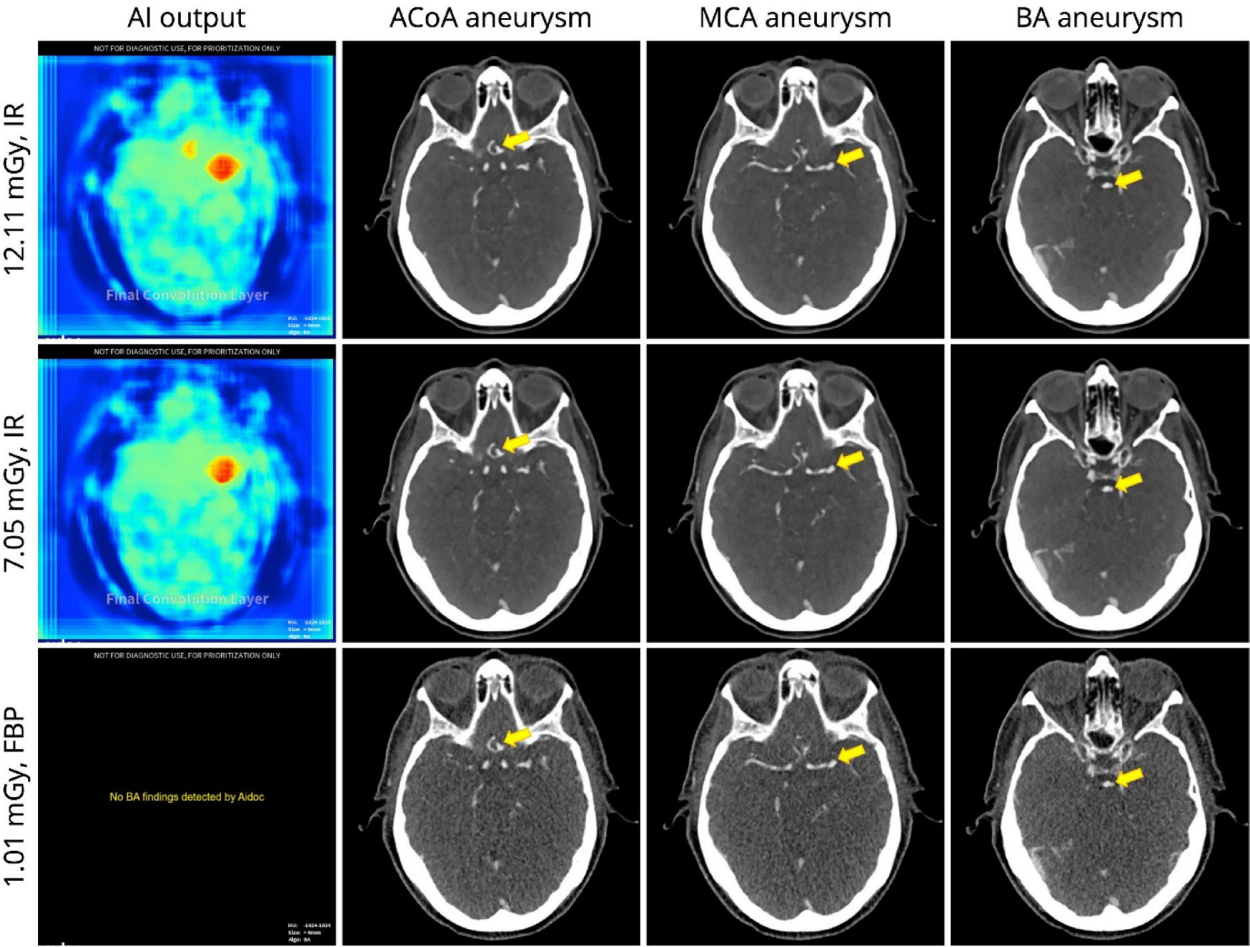
Examples of AI output and corresponding CT images. First row: The AI-generated heatmap displays true positive labels for aneurysms in the anterior communicating artery (ACoA) and middle cerebral artery (MCA), but not for the basilar artery (BA). Second row: The heatmap indicates a true positive label for the MCA aneurysm, but not for the ACoA and BA aneurysms. Third row: A false negative summary report generated by the AI solution, where BA stands for brain aneurysm. IR = iterative reconstruction, FBP = filtered back projection.

The orchestrator processed all scans, AI results (positive findings on heatmaps or negative summary reports) were obtained without exception. Out of the 126 scans, 117 (92.9%) generated heatmaps that implied true positive triage decisions, while 9 (7.1%) resulted in negative outputs corresponding to false negative triage decisions. Figure 2 shows examples of phantom scans and AI output. A scan data set covering all doses and image reconstructions investigated in this study is available at the following link: https://kaggle.com/datasets/6268f60ee63bda9b513121e3c088d73982476a1eb96ed5028da2bdf79273675b.

### AI labeling of the MCA, ACoA, and BA aneurysms

**Fig. 3:**
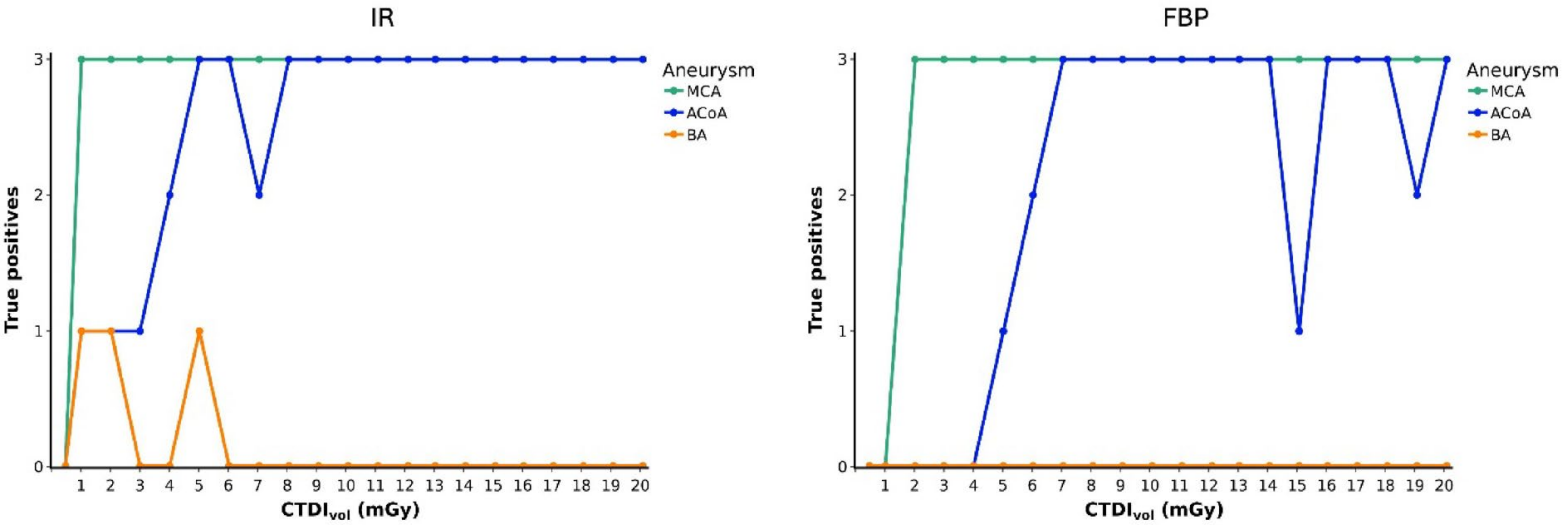
True positive aneurysm labels in heatmaps generated by the AI solution. Graphs display the absolute numbers of true positive aneurysm labels across three repeated CT examinations per dose and image reconstruction method. IR = iterative reconstruction, FBP = filtered back projection, MCA = middle cerebral artery, ACoA = anterior communicating artery, BA = basilar artery, CTDI_vol_ = computed tomography dose index.

**Fig. 4:**
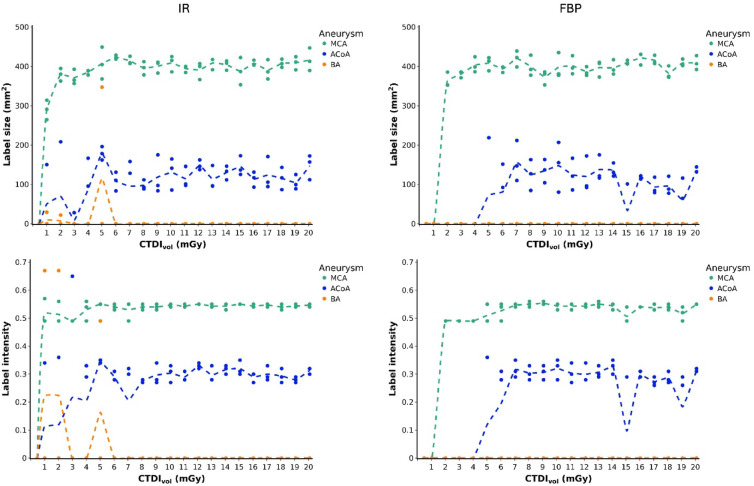
Sizes and Intensities of aneurysm labels. Graphs present the size (upper row) and intensity (lower row) of aneurysm labels generated by the AI solution in three repeated CT examinations per dose and image reconstruction method. Dotted lines indicate mean values. IR = iterative reconstruction, FBP = filtered back projection, MCA = middle cerebral artery, ACoA = anterior communicating artery, BA = basilar artery, CTDI_vol_ = computed tomography dose index.

Overall, the AI solution generated 214 heat map labels. Review of the labels by two radiologists yielded no false positive intracranial aneurysm finding and no disagreement between the two readers. Figure 3 presents true positive aneurysm labels per aneurysm. Label sizes and intensities are detailed in Fig. 4. Across all 126 acquisitions, the MCA aneurysm was most frequently labeled (117 labels, 92.9%), followed by the ACoA aneurysm (94 labels, 74.6%) and the BA aneurysm (3 labels, 2.4%). Also, the size and intensity of MCA labels were greater than those of ACoA and BA labels (*p* < 0.001) and they were associated with better reproducibility. CVs for label size were 0.08 (MCA) and 0.25 (ACoA) with IR and 0.05 (MCA) and 0.26 (ACoA) with FBP. CVs for label intensity were 0.04 (MCA) and 0.08 (ACoA) with IR and 0.05 (MCA) and 0.08 (ACoA) with FBP.

### AI aneurysm labeling in IR-reconstructed acquisitions

**Fig. 5:**
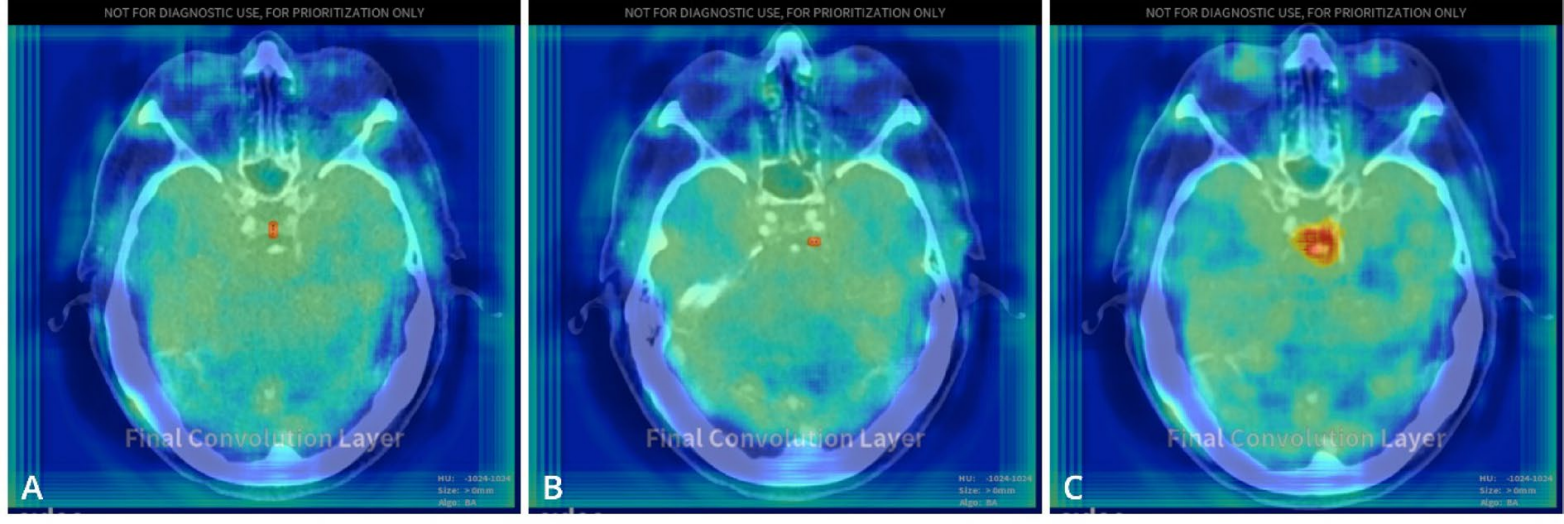
Heatmaps generated by the AI solution with positive labels for the basilar artery aneurysm superimposed on corresponding CT images. Labels in CT examinations at 1.01 mGy (**A**) and 2.02 mGy (**B**) are slightly offset. (**C**) The label in a CT examination at 5.02 mGy aligns with the aneurysm. All CT examinations presented were reconstructed using iterative reconstruction.

Using the department’s standard image reconstruction algorithm, IR, doses of ≥ 8 mGy resulted in correct and reproducible labeling of the MCA and ACoA aneurysms. In contrast, ACoA aneurysm labels were not reproducible at doses below 8 mGy, except at 5 and 6 mGy. Interestingly, the only acquisition that produced true positive labels for all three aneurysms present in the phantom occurred at 5 mGy with IR. However, this result was non-reproducible as the BA aneurysm was labeled in only one of three repeated acquisitions. Incidental BA labels were also produced at 1 and 2 mGy, but these labels were small and slightly offset when superimposed with the original scan data (Fig. 5). The lowest dose of 0.5 mGy produced no aneurysm labels, while the MCA label was robust at higher doses. False positive vessel occlusion alerts were generated at doses of 3 mGy in 1 of 3 repetitions and 2 mGy in 2 of 3 repetitions.

### AI aneurysm labeling in FBP-reconstructed acquisitions

Using FBP for image reconstruction resulted in decreased AI labeling of the MCA (57 vs. 60 with IR), ACoA (42 vs. 52 with IR), and BA (0 vs. 3 with IR) aneurysms. ACoA aneurysm labeling deteriorated at doses below 7 mGy, similar to IR. However, unlike with IR, ACoA labeling was also non-reproducible at higher doses of 15 and 19 mGy. None of the aneurysms were labeled at 0.5 mGy, as with IR, and additionally at 1 mGy. A false positive vessel occlusion alert was generated at 2 mGy in 1 of 3 repetitions.

### Reader assessment

**Fig. 6:**
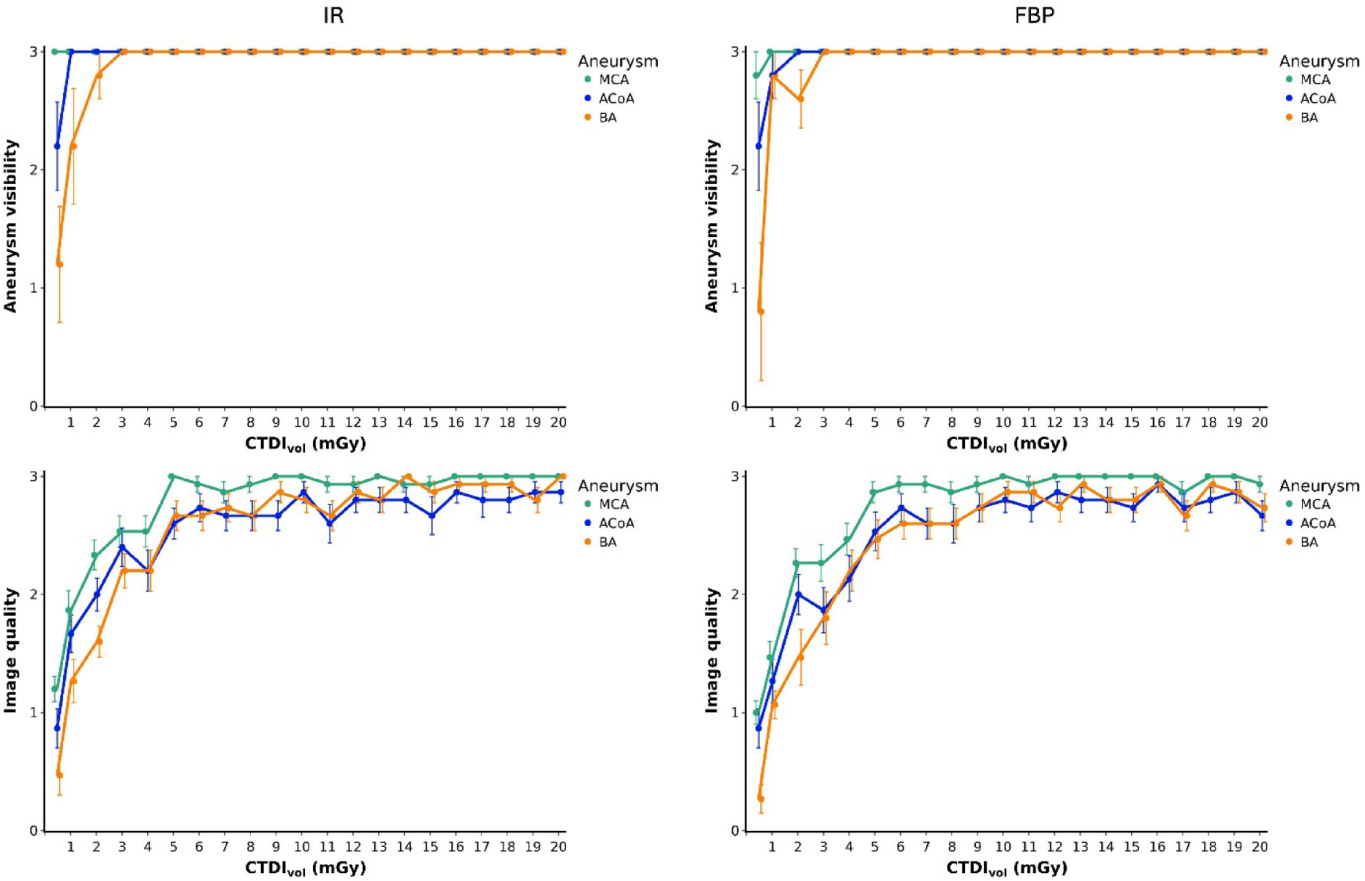
Results of aneurysm assessment by five neuroradiologists. Upper row: Absolute number of CT examinations in which aneurysms were rated as visible across three repeated CT examinations per dose and image reconstruction method. Lower row: Image quality ratings per aneurysm, dose, and image reconstruction method. Mean results of the five readers are presented; error bars indicate standard errors of the mean. IR = iterative reconstruction, FBP = filtered back projection, MCA = middle cerebral artery, ACoA = anterior communicating artery, BA = basilar artery, CTDI_vol_ = computed tomography dose index.

Figure 6 presents the results of the reading experiment. Numerical results are provided in supplementary Tables 1 and 2. Readers found at least one aneurysm visible in 100% of cases. With the exception of one reader and one FBP-reconstructed scan at 0.47 mGy, readers always found the MCA aneurysm visible (629/630 positive responses, 99.8%). The ACoA aneurysm was rated to be visible in 98.6% of cases (621/630), and the BA aneurysm in 95.4% of cases (601/630). 100% of aneurysms were found to be visible at doses > 2 mGy, regardless of image reconstruction, while doses ≤ 2 mGy degraded aneurysm visibility in both IR- and FBP-reconstructed images. Image quality ratings deteriorated at lower doses in both IR and FBP reconstructions. Image quality ratings for the MCA aneurysm were superior to those for ACoA and BA aneurysms in both IR (*p* ≤ 0.038) and FBP (*p* ≤ 0.012), whereas no difference was found between the ACoA and BA aneurysms regardless of the image reconstruction method (*p* ≥ 0.893). There was also no difference in image quality ratings between the two reconstruction methods for any of the three aneurysms (*p* = 1).

## Discussion

Scanner-related changes in data quality are common in medical imaging, yet monitoring their impact on diagnostic AI performance remains challenging. In this study, we performed standardized consistency testing of a commercial AI solution for intracranial aneurysm detection across changes in CT data quality caused by dose and image reconstruction. The assessment was based on a phantom replicating a patient with aneurysms in the anterior communicating artery (ACoA), middle cerebral artery (MCA), and basilar artery (BA). Our findings reveal differences between aneurysm types, with only 3 BA labels (2.4%) versus 94 (74.6%) for the ACoA and 117 (92.9%) for the MCA. The AI maintained stable performance within the medium dose range but generated inconsistent results at doses below 8 mGy with iterative reconstruction and at doses below 7 mGy and above 14 mGy with filtered back projection (FBP). Neuroradiologists were less affected by variations in data quality and reported 100% visibility of all three aneurysms at doses above 2 mGy.

Diagnostic AI solutions are developed and authorized using datasets that are representative of the target population to ensure adequate generalizability for clinical application^21^. Naturally, these datasets tend to include more common conditions, while rarer cases are less represented. While no specific details are disclosed regarding the training data of the AI algorithm we assessed, it is plausible that the AI lacked sufficient exposure to images with low or high radiation doses and FBP, which would explain its reduced performance under these conditions. This issue was particularly evident in the ACoA aneurysm, which was located in a more challenging position near the bone at the base of the skull, leading to lower network attention compared to the MCA aneurysm.

Furthermore, although no size-based restriction was applied in the clinical workflow, the FDA’s original clearance document specifies a limitation to aneurysms larger than 5 mm^19^. Assuming this reflects a lower training prevalence of smaller aneurysms, our results show that the algorithm still performs accurately for 4-mm aneurysms within the typical clinical dose range. However, performance degradation outside these parameters may have been exacerbated by the aneurysm size falling slightly below the algorithm’s expected range. Similarly, the algorithm’s lack of response to the 2-mm BA aneurysm may reflect both a low prevalence of this aneurysm type in the training data and the general rarity of BA aneurysms in the population, compounded by the previously reported challenges AI systems face in detecting very small aneurysms^22^.

In light of the above, our results indicate that while the AI performs well under conditions that match its learned parameters, its performance degrades when exposed to conditions outside its expected input domain. As a result, seemingly non-critical changes to the imaging environment can lead to unexpected AI performance^23^. For instance, a routine software update to mammography systems was previously reported to cause an erroneous increase in AI-generated recall rates during breast cancer screening^12^. Our findings demonstrate similar unexpected AI behavior, with inconsistent responses triggered by dose increase with FBP, which contrasts with the conventional expectation that higher doses generally improve diagnostic image quality for radiologists.

These insights carry practical implications for radiological practice. AI users are often unaware of the limitations a particular algorithm may have in their specific setting. Given the apparent disconnect between AI behavior and radiological conventions, changes to the imaging environment can unpredictably alter AI performance. As a result, radiologists should exercise caution when adapting imaging technologies solely based on their own needs if AI tools are in use, and any such changes should be followed by rigorous revalidation to ensure continued algorithm reliability^23^. This requires consistency testing, which is also relevant beyond intended modifications of imaging equipment to monitor and detect AI decay^13,23^.

Current consistency test concepts rely on monitoring AI during clinical routine, meaning inconsistent AI performance becomes apparent if the AI output does not align with radiologists’ assessments^24^. This process offers limited testing opportunities, lacks objectivity and standardization, and its retrospective nature potentially jeopardizes patients exposed to inadequate AI performance. Phantoms are standard instruments for prospective consistency testing but have not been applicable for AI due to their lack of anatomical and pathological features. Dedicated phantoms with realistic features have therefore been defined as a key element for validation and quality control in imaging and AI ecosystems^25^. In this study, we employed such a phantom to demonstrate the impact on AI when modifications to imaging equipment are made.

Performing the same evaluation in patients would not be feasible due to ethical and practical constraints. Specifically, repeated scans of individual patients for consistency testing are not possible, which precludes the data access and standardization necessary for our analysis. Even with a larger prospective cohort, systematic dose variation would be ethically unjustifiable, and clinical variability would prevent the comparability required for standardized testing. Retrospective collection of patient data would face the same variability issues and require multi-institutional coordination to identify suitable cases, which would undermine the goal of assessing local AI performance on a specific scanner system. Additionally, the AI setup precluded any possibility of retrospective data analysis with the system.

Our study had limitations. First, a comparative analysis of different AI applications across multiple scanners was beyond the scope of this work. Likewise, a comprehensive assessment of AI across the biological variability of aneurysms and human anatomy was not our aim. Second, due to the closed nature of the AI solution and lack of quality control interfaces, we were limited to standard AI heatmap output and cannot provide proof that missing AI responses were associated with false negative triage decisions. Third, unlike the AI, the participating neuroradiologists were able to learn from our data, which prevented us from assigning them the exact same search and detection task as the AI. Instead, they performed a visual grading analysis, which inherently involves subjectivity. Lastly, as repetitive and standardized testing in patients is precluded, we were not able to conduct the same testing in a patient cohort as outlined above.

In conclusion, diagnostic AI for intracranial aneurysm detection performs inconsistently when CT image quality is not optimal and shows less resilience to data quality changes than radiologists. The AI response does not follow conventional relationships between dose, image quality, and diagnostic performance. Our findings highlight the need for standardized monitoring of AI performance, and quality control interfaces in AI solutions would facilitate this process.

## Electronic supplementary material

Below is the link to the electronic supplementary material.

Supplementary Material 1.

## Data Availability

Scan data analyzed in this study is available at the following link: https://kaggle.com/datasets/6268f60ee63bda9b513121e3c088d73982476a1eb96ed5028da2bdf79273675b.

## References

[1] Rinkel, G. J., Djibuti, M., Algra, A. & van Gijn, J. Prevalence and risk of rupture of intracranial aneurysms: A systematic review. *Stroke* **29**, 251–256. 10.1161/01.str.29.1.251 (1998). 9445359 10.1161/01.str.29.1.251

[2] Vlak, M. H., Algra, A., Brandenburg, R. & Rinkel, G. J. Prevalence of unruptured intracranial aneurysms, with emphasis on sex, age, comorbidity, country, and time period: A systematic review and meta-analysis. *Lancet Neurol.* **10**, 626–636. 10.1016/S1474-4422(11)70109-0 (2011). 21641282 10.1016/S1474-4422(11)70109-0

[3] Vernooij, M. W. et al. Incidental findings on brain MRI in the general population. *N Engl. J. Med.* **357**, 1821–1828. 10.1056/NEJMoa070972 (2007). 17978290 10.1056/NEJMoa070972

[4] Westerlaan, H. E. et al. Intracranial aneurysms in patients with subarachnoid hemorrhage: CT angiography as a primary examination tool for diagnosis–systematic review and meta-analysis. *Radiology* **258**, 134–145. 10.1148/radiol.10092373 (2011). 20935079 10.1148/radiol.10092373

[5] Yoon, N. K., McNally, S., Taussky, P. & Park, M. S. Imaging of cerebral aneurysms: A clinical perspective. *Neurovascular Imaging* **2**, 6. 10.1186/s40809-016-0016-3 (2016).

[6] Park, A. et al. Deep learning-assisted diagnosis of cerebral aneurysms using the HeadXNet model. *JAMA Netw. Open.* **2**, e195600. 10.1001/jamanetworkopen.2019.5600 (2019). 31173130 10.1001/jamanetworkopen.2019.5600 PMC6563570

[7] Yang, J. et al. Deep learning for detecting cerebral aneurysms with CT angiography. *Radiology* **298**, 155–163. 10.1148/radiol.2020192154 (2021). 33141003 10.1148/radiol.2020192154

[8] Allen, B. et al. Evaluation and Real-World performance monitoring of artificial intelligence models in clinical practice: Try it, buy it, check it. *J. Am. Coll. Radiol.* **18**, 1489–1496. 10.1016/j.jacr.2021.08.022 (2021). 34599876 10.1016/j.jacr.2021.08.022

[9] Rajpurkar, P. & Lungren, M. P. The current and future state of AI interpretation of medical images. *N Engl. J. Med.* **388**, 1981–1990. 10.1056/NEJMra2301725 (2023). 37224199 10.1056/NEJMra2301725

[10] You, W. et al. Protocol and preliminary results of the establishment of intracranial aneurysm database for artificial intelligence application based on CTA images. *Front. Neurol.* **13**, 932933. 10.3389/fneur.2022.932933 (2022). 35928124 10.3389/fneur.2022.932933 PMC9343848

[11] Yu, A. C., Mohajer, B. & Eng, J. External validation of deep learning algorithms for radiologic diagnosis: A systematic review. *Radiology: Artif. Intell.* **4**, e210064. 10.1148/ryai.210064 (2022). 10.1148/ryai.210064 PMC9152694 35652114

[12] de Vries, C. F. et al. Impact of different mammography systems on artificial intelligence performance in breast cancer screening. *Radiol. Artif. Intell.* **5**, e220146. 10.1148/ryai.220146 (2023). 37293340 10.1148/ryai.220146 PMC10245180

[13] Feng, J. et al. Clinical artificial intelligence quality improvement: towards continual monitoring and updating of AI algorithms in healthcare. *NPJ Digit. Med.* **5**, 66. 10.1038/s41746-022-00611-y (2022). 35641814 10.1038/s41746-022-00611-y PMC9156743

[14] Ross, J., Hammouche, S., Chen, Y. & Rockall, A. G. Royal college of radiologists, A. I. W. G. Beyond regulatory compliance: Evaluating radiology artificial intelligence applications in deployment. *Clin. Radiol.* **79**, 338–345. 10.1016/j.crad.2024.01.026 (2024). 38360516 10.1016/j.crad.2024.01.026

[15] Jahnke, P. et al. Radiopaque three-dimensional printing: A method to create realistic CT phantoms. *Radiology* **282**, 569–575. 10.1148/radiol.2016152710 (2017). 27626676 10.1148/radiol.2016152710

[16] Jahnke, P. et al. Paper-based 3D printing of anthropomorphic CT phantoms: Feasibility of two construction techniques. *Eur. Radiol.* **29**, 1384–1390. 10.1007/s00330-018-5654-1 (2019). 30116957 10.1007/s00330-018-5654-1

[17] Ardila Pardo, G. L. et al. 3D printing of anatomically realistic phantoms with detection tasks to assess the diagnostic performance of CT images. *Eur. Radiol.* **30**, 4557–4563. 10.1007/s00330-020-06808-7 (2020). 32221686 10.1007/s00330-020-06808-7 PMC7338819

[18] Michallek, F., Genske, U., Niehues, S. M., Hamm, B. & Jahnke, P. Deep learning reconstruction improves radiomics feature stability and discriminative power in abdominal CT imaging: A phantom study. *Eur. Radiol.* **32**, 4587–4595. 10.1007/s00330-022-08592-y (2022). 35174400 10.1007/s00330-022-08592-y PMC9213380

[19] U.S. Food and Drug Administration. 510(k) Premarket Notification (2022). https://www.accessdata.fda.gov/cdrh_docs/pdf21/K213721.pdf (Accessed Apr 20 2025).

[20] Genske, U. & Jahnke, P. Human observer net: A platform tool for human observer studies of image data. *Radiology* **303**, 524–530. 10.1148/radiol.211832 (2022). 35258375 10.1148/radiol.211832

[21] Chalkidou, A. et al. Recommendations for the development and use of imaging test sets to investigate the test performance of artificial intelligence in health screening. *Lancet Digit. Health*. **4**, e899–e905. 10.1016/S2589-7500(22)00186-8 (2022). 36427951 10.1016/S2589-7500(22)00186-8

[22] Bizjak, Z. & Spiclin, Z. A. Systematic review of deep-learning methods for intracranial aneurysm detection in CT angiography. *Biomedicines* **11**10.3390/biomedicines11112921 (2023). 10.3390/biomedicines11112921 PMC10669551 38001922

[23] Brady, A. P. et al. Developing, purchasing, implementing and monitoring AI tools in radiology: Practical considerations. A multi-society statement from the ACR, CAR, ESR, RANZCR and RSNA. *Radiol. Artif. Intell.* **6**, e230513. 10.1148/ryai.230513 (2024). 38251899 10.1148/ryai.230513 PMC10831521

[24] Daye, D. et al. Implementation of clinical artificial intelligence in radiology: Who decides and how? *Radiology* **305**, 555–563. 10.1148/radiol.212151 (2022). 35916673 10.1148/radiol.212151 PMC9713445

[25] Wang, G. et al. Development of metaverse for intelligent healthcare. *Nat. Mach. Intell.* **4**, 922–929. 10.1038/s42256-022-00549-6 (2022). 36935774 10.1038/s42256-022-00549-6 PMC10015955

